# The effect of exercise on the quality of life in an academic environment

**DOI:** 10.1038/s41598-023-29650-5

**Published:** 2023-02-11

**Authors:** Arwa Alumran

**Affiliations:** Health Information Management and Technology Department, College of Public Health, Imam Abdulrahman Bin Faisal University, Dammam, Saudi Arabia

**Keywords:** Health care, Risk factors

## Abstract

Regular physical activity has a direct association with an improvement in perceived health-related quality of life (HRQL). Because many Saudis are reportedly inactive, Imam Abdulrahman bin Faisal University established a walking challenge for all university employees to encourage a better lifestyle and to promote health awareness. This study aims to measure the differences in the participants’ HRQL scores before and after the challenge. A before and after study was conducted using HRQL survey that was sent to all university employees before the implementation of a pedometer-based walking challenge at the study setting, and after the challenge ended. A randomized snowball sample method was used to recruit participants. The differences in the overall HRQL before and after the challenge were calculated. A RAND SF20-items scale was used to measure the participants’ HRQL scores, along with other information such as academic qualifications. Most of the participants were between 31 and 50 years old, and 40% were males. There was a statistically significant difference in the overall HRQL scores before and after the walking challenge intervention. The HRQL score increased from 50.77 before the challenge to 55.53 after the challenge (paired *t-*test = − 4.322, *P* < 0.0001). An odds ratio (OR) showed that the odds of having higher HRQL scores increased by 88% after the walking challenge, compared to before the walking challenge (OR = 1.88; 95% CI = 1.269–2.809; *P* = 0.002). Physical activity by itself can improve a community’s overall health and quality of life. Similar interventions are encouraged in all public and private sectors in the country.

## Introduction

Health-related Quality of life (HRQL) is defined by the World Health Organization as “the individual’s perception of their position in life in the context of culture and value systems in which they live, and in relation to their goals, expectations, standards, and concerns”^1,2^. Health-related quality of life (HRQL) is influenced by many constructs, such as work environment, living environment, physical and psychological health, and social life^3^. Some researchers associate HRQL with overall satisfaction with life, which can be measured through various domains (e.g., physical, cognitive, emotional, and social well-being)^4,5^. In addition, Krein, *et. al.*^6^ and Zheng, *et. al.*^7^ linked lower quality of life with chronic pains and conditions, which associated to an increased burden on healthcare services.

Sufficient worldwide evidence has demonstrated the direct association of regular physical activity with an improvement in perceived HRQL^8–11^. In addition to the improvement in HRQL, increasing amounts of physical activity has been found to be directly associated with decreased overall mortality and better community health^9,12^. However, many Saudis do not engage in regular exercise or walking, and 60% of Saudis are considered physically inactive according to national and international reports^13–15^. Assessing the association between physical activity and HRQL is an area that needs further investigation in Saudi Arabia.

Furthermore, in a comprehensive meta-analysis conducted by Gillison, *et. al.*^16^ the authors found that a meaningful improvement in HRQL can be achieved using exercise interventions in healthy populations. Similarly, Moy, *et. al.*^17^ believe that complying with a healthy steps routine improves the person’s HRQL. The link between exercise and HRQL in Eastern Saudi Arabia needs to be assessed.

The Quality-of-Life Program was established in Saudi Arabia in 2018 as part of Vision 2030’s Vision Realizations Programs. The program has four main objectives, one of which is “to increase public participation in sports and athletic activities”^18,19^.

Imam Abdulrahman bin Faisal University (IAU) in Dammam, Saudi Arabia, started a walking challenge as a part of the Vision 2030 Realization Program to increase the health awareness of its employees and their families. Such intervention programs can increase the awareness of health risks associated with inactive lifestyles and thus motivate physical activity and a healthier lifestyle^20–22^. Assessing HRQL before and after a real-life physical activity intervention can provide clear evidence of a causal association and thus promote exercise as a way of improving HRQL.

The evidence of a positive association between increased physical activity and the enhancement of HRQL can provide the population with the right incentive to be more physically active^23,24^. This meaningful, motivational public health approach is expected to result in less mortality and a decrease in the burden of disease in the country.

Although many studies around the world have measured and confirmed the association of physical activity with the HRQL scores^25–27^, studies in this area are scarce in Saudi Arabia. Therefore, the aim of this present study is to assess the efficacy of exercise interventions in improving HRQL scores in an academic institute in Saudi Arabia. The research hypothesis is that walking improves the person’s HRQL scores.

## Methods

The aim of this study is to measure the effect of a pedometer-based walking challenge on the HRQL. The challenge was introduced in Imam Abdulrahman bin Faisal University (IAU) and all university employees were asked to join the challenge.

The challenge started on the first of March 2019 and ended on 31st March 2019. The organizers chose this date so the weather would be moderate during the spring. Participants were asked to use a specific free mobile-based pedometer application^28^, through registration to the university pool, so the organizers can follow up on the progress of the participants. Pacer app was selected because of its high rating among users in different operating systems and was reported by researchers as the most attractive^29^. According to Bassett, *et. al.*^30^, the Pacer application is highly accurate.

Non-probability snowball sampling technique was applied to recruit the required participants. An online HRQL survey was sent to all employees (administrative and faculty) before the challenge started. Completing the survey was entirely optional, and the participants information were only used for research purposes as mentioned to the participants in the survey; participants could leave the survey at any time. The survey was sent again after the challenge ended to the same targeted group. The survey is in an accordance with the CHERRIES checklist for e-surveys^31^.

The total number of employees in IAU is approximately 8000 individuals. Of those, only 1257 employees joined the challenge. The estimated sample size was 294, according to the Cochran^32^ method.

An email including an online survey of The RAND SF-20-items scale^33^ was sent to members who joined the challenged and requested them to complete the scale, to measure their HRQL. SF-36 is the wider scope survey which is recommended by several studies^5,34,35^, however, SF2-0 is used to ensure complete responses.

The email was sent through the university’s public relations office before the challenge started. Two-hundred and eight participants completed the survey. It’s hard to calculate the response rate as the questionnaire was distributed online. The survey was sent after the challenge ended and was completed by 200 participants.

Ethical approval was obtained from the Institutional Review Board of Imam Abdulrahman bin Faisal University to conduct this study and all methods were performed in accordance with the relevant guidelines and regulations of the institution. Informed Consent was obtained from participants before completing the survey.

Univariate analysis was conducted to measure the participants characteristics. Followed by bivariate analyses to assess the outcome variable (HRQL) before and after the pedometer-based walking challenge. Further, other variables were tested to assess their association with the outcome variable to rule out any confounding effects. and the variables in the study to find out any relationship with the outcome (e.g., education, position, … etc.). Chi-square and Analysis of Variance (ANOVA) were used to conduct the bivariate analyses. Further, odds ratio was calculated to measure the association between the high/low HRQL and before/after the challenge using binary logistic regression.

### Ethics approval and consent to participate

Ethical approval was obtained from the Institutional Review Board of Imam Abdulrahman bin Faisal University to conduct this study and all methods were performed in accordance with the relevant guidelines and regulations of the institution.

### Informed Consent

Informed Consent was obtained from participants.

## Results

The majority of respondents were females (*n* = 124, 60%). More than half of the participants were Saudis (*n* = 130, 63%). More than 70% of the respondents were from the age groups 31 to 40 years old (*n* = 96, 46%) and 41 to 50 years old (*n* = 66, 32%). Most of the participants have a Bachelor’s degree (*n* = 76, 37%), followed by PhD degree holders (*n* = 60, 29%). The majority of participants were administrative officers (*n* = 89, 43%), followed by lecturers and assistant professors (*n* = 49, 34%, each). And only 5.3% of participants were associate professor or full professors (Table 1).

**Table 1:** Mean HRQL Score Across Study Participants.

Variable	Frequency (%) n = 208	Mean HRQL (SD)	Mean difference	Test (*P*-Value)
Language				
Arabic	150 (72.1)	50.23 (11.73)		
English	58 (27.9)	52.17 (12.13)	–1.946	*t* = − 1.063 (.289)
Gender				
Male	83 (39.9)	52.55 (10.77)		
Female	124 (59.6)	49.78 (12.26)	2.772	*t* = 1.672 (.096)
Missing	1 (.5)	–		
Nationality				
Saudi	130 (62.5)	49.15 (12.39)		
Non-Saudi	78 (37.5)	53.46 (10.418)	− 4.308	*t* = − 2.686* (.008
Age				
21–30 years old	27 (13.0)	52.67 (11.37)	NA^†^	*f* = 2.238 (.066)
31–40 years old	96 (46.2)	48.16 (12.07)		
41–50 years old	66 (31.7)	52.98 (11.30)		
51–60 years old	15 (7.2)	53.53 (11.48)		
> 60 years old	4 (1.9)	53.75 (12.55)		
Education				
Secondary or high school	15 (7.2)	48.87 (13.37)	NA^†^	*f* = 0.483 (.694)
Bachelor’s degree	76 (36.5)	49.97 (10.37)		
Master’s degree	56 (26.9)	51.36 (13.69)		
PhD degree	60 (28.8)	51.95 (11.46)		
Missing	1 (0.5)	–		
Position				
Administrative officer	89 (42.8)	48.94 (11.83)	NA^†^	*f* = 968 (.438)
Demonstrator	10 (4.8)	51.20 (8.83)		
Lecturer	49 (23.6)	52.98 (11.85)		
Assistant professor	49 (23.6)	51.31 (12.25)		
Associate professor	6 (2.9)	55.33 (15.32)		
Full professor	5 (2.4)	50.00 (7.97)		

*p < 0.05.

^†^NA: the variable has more than 2 groups, thus measuring the mean difference is not applicable.

*HRQL* Health-related quality of life.

The internal consistency of the scale has shown to be reliable (Cronbach’s α = 0.853)^36^, and the scale’s validity has been tested in other studies^33,37^.

Bivariate analysis showed no statistically significant difference in any of the independent variables in the HRQL scores, except for nationality (*t* = − 2.686*, P = 0.008). Non-Saudis exhibited higher HRQL scores compared to Saudis (mean difference = 4.308).

Although gender is not significantly associated with the HRQL in the current study, however, males in study have a better mean HRQL score compared to females (mean difference = 2.772). In addition, participants in the age group 31–40 years old have the lowest average HRQL score compared to other age groups in the study (mean HRQL_31-40_ = 48.16, SD = 12.07).

Furthermore, participants with administrative jobs have lower average HRQL compared to academics (mean HRQL _administrative officer_ = 48.94, SD = 11.83), followed by full professors (mean HRQL _full professor_ = 50, SD = 7.97). while the highest average HRQL according to the position is for associate professors (mean HRQL _associate professor_ = 55.33, SD = 15.32).

When the HRQL is compared across different degrees, the highest average HRQL scores is seen in PhD degree holders (mean HRQL _PhD degree_ = 51.95, SD = 11.46). While secondary or high school degree holders have a mean HRQL score of 48.87 (SD = 13.37).

**Figure.1:**
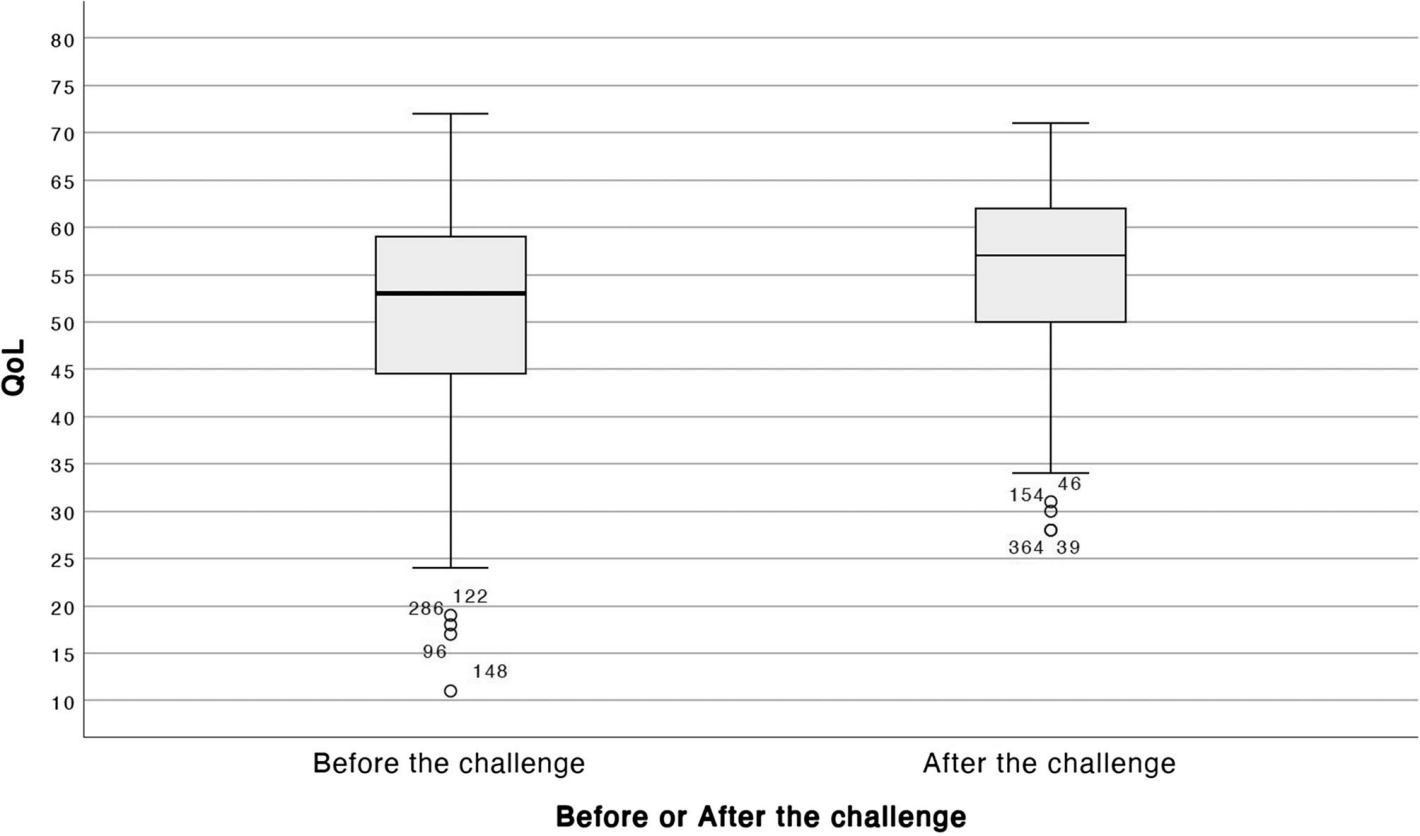
Overall HRQL Score Before and After the Walking Challenge.

The study’s results revealed a statistically significant difference in overall HRQL for employees of Imam Abdulrahman bin Faisal University after the walking challenge intervention. The HRQL score increased from 50.77 (*SD* = 11.85) before the challenge to 55.53 (*SD* = 9.25) after the challenge (mean difference = -4.76), and this result was statistically significant (Paired *t-*test = -4.322) at a 0.0001 level of significance (95% CI = -6.96, -2.60) (Fig. 1, and Table 2).

**Table 2:** The HQOL score before and after the challenge.

t	df	*P*-Value	Mean Difference	95% CI of the difference	
				Lower	Upper
− 4.529	389.762	.000	− 4.756	− 6.820	− 2.691

DV: HQOL score / IV: before and after the walking challenge.

The participants in the study were grouped at the analysis level into low HRQL score and high HRQL score; those who scored above the mean had a high score, and those who scored below the mean were considered to have a low score. A chi-square test showed a significant association between the HRQL scores and the effect of the walking challenge (*x*^2^ = 9.924, *P* = 0.002). An odds ratio (OR) showed that the odds of having higher HRQL scores increased by 88% after the walking challenge, compared to before the walking challenge (OR = 1.88; 95% CI = 1.269–2.809; *P* = 0.002).

## Discussion

Physical activity was not actively promoted in Saudi Arabia prior to the announcement of Vision 2030^19^. Therefore, in accordance with the Kingdom’s new Vision 2030, Imam Abdulrahman bin Faisal University in Dammam, Saudi Arabia, started a walking challenge to promote physical activity for all university employees. To the author’s knowledge, this is the first study to assess HRQL scores in university employees before and after a walking challenge, which is considered an intervention in this study.

The data reported in this study showed that the mean HRQL score in this current study was significantly different before and after the walking intervention. The mean HRQL was higher after the walking challenge. This study’s results are consistent with those of other studies, including a recent systematic review and meta-analyses, which all confirm that regular physical activity enhances HRQL^16,23,25,38,39^. Similarly, Puciato, Rozpara, and Borysiuk^40^ indicated that in their study which was conducted in Poland, physically active respondent had the highest level of HRQL score. Hence, regular physical activity enhances general health and well-being and improves a person’s overall HRQL.

On the contrary, a study conducted by Lustyk, et. al^41^ in the United States of America found that only high frequency exercise significantly improves the participants HRQL, compared to low intensity, such as walking. This is conflicting results to what this study found, where the main physical activity measured was walking. This difference could be due to different environmental and habitual variables between the two communities, i.e., USA and Saudi Arabia.

In addition, the current study also showed a significant increase in the odds ratio of having a high HRQL score after the challenge compared to the low HRQL before the challenge. Similarly, a large study conducted by Brown, *et. al.*^42^ showed that the odds of active subjects reporting at least 14 unhealthy days in the last month is half that of the inactive subjects. This evidence suggests a positive association between HRQL and physical activity.

Although females in the study have lower HRQL scores compared to males, which is consistent with the results from other studies^43,44^, however, this difference was not found significant in the current study. None of the demographic characteristics in the study participants influenced their HRQL score significantly, except their nationality which could be due to the unequal distribution of Saudis vs non-Saudis in the study.

The subjectivity of participants plays a major role in the assessment of their HRQL, specifically regarding their physical, emotional, and social well-being^45^. Thus, one of the limitations of this study is the self-reported HRQL data, which may lead to an over- or underestimation of HRQL scores. However, this is the only existing reliable method for measuring HRQL score^45^. In addition, given this study’s focus on university employees, the study sample included academic institution employees, which ultimately impacts the generalizability of the results to the general population.

Further studies are needed to assess the dose–response association between physical activity and HRQL, and the influence of the type of physical activity on the HRQL (i.e., the effect of certain types of physical activity on the improvement on HRQL scores), specifically in the Saudi community.

This study encourages other academic institutes in Saudi Arabia to conduct similar physical activity challenges following the footsteps of Imam Abdulrahman bin Faisal University. Encouraging the community to be more physically active is recommended, not only to enhance overall HRQL but also to enhance the overall health and well-being of the population.

## Conclusion

The main objective of this study is to assess the influence of the walking challenge on the improvement of the university employees’ HRQL. The study showed a positive association between the walking challenge and the improvement of the HRQL scores in the study population. In other words, high physical activity is positively correlated with an improvement in HRQL. The results of this study could be the basis for further similar interventional studies across the country to enhance the overall health and well-being of the Saudi population.

This study is aligned with Saudi Vision 2030^19^. More academic institutes in Saudi Arabia are encouraged to conduct similar challenges to gain an overall healthier community with lower chronic diseases and lower mortality levels.

## Data Availability

The datasets used and/or analyzed during the current study are available from the corresponding author on reasonable request.

## Abbreviations

WHO: World Health Organization
HRQL: Health-related quality of life
IAU: Imam Abdulrahman bin Faisal University
ANOVA: Analysis of Variance
